# Dengue dynamics beyond biological factors: Revealing the nexus between urbanisation planning, and mobilities in Vientiane, Lao PDR

**DOI:** 10.1371/journal.pntd.0011990

**Published:** 2025-06-16

**Authors:** Olivier Telle, Marc Grandadam, Damien Philippon, Elodie Calvez, Virginie Pommelet, Sebastien Marcombe, Josephin Béraud, Somphavanh Somlor, Marc Choisy

**Affiliations:** 1 Institut de Recherche sur l’Asie du Sud-Est Contemporaine (IRASEC), CNRS, Bangkok, Thailand; 2 IRL HealthDEEP, CNRS, Bangkok, Thailand; 3 Arbovirus and Emerging Viral Diseases Laboratory, Institut Pasteur du Lao PDR, Vientiane, Laos; 4 Institut de Recherche Biomedicale des Armees Bretigny sur Orge; 5 Medical Entomology & Biology of Disease Vectors, Institut Pasteur du Laos, Laos, France; 6 Vector Control Consulting - South East Asia (VCC-SEA), Vientiane, Laos; 7 Centre National de la Recherche Scientifique (CNRS@Create), Singapore; 8 Centre for Tropical Medicine and Global Health, Nuffield Department of Clinical Medicine, University of Oxford, Oxford, United Kingdom; 9 Oxford University Clinical Research Unit, Ho Chi Minh City, Vietnam; Huazhong University of Science and Technology Tongji Medical College, CHINA

## Abstract

**Background:**

Dengue fever, a vector-borne disease transmitted by *Aedes* mosquitoes, poses a significant public health challenge in urban Southeast Asia. While urbanisation is widely recognised as a driver of dengue transmission, its effects are multifaceted, creating both risks and protective factors. Despite its longstanding presence in Laos, limited research has explored the geographic and epidemiological dynamics of dengue in Vientiane, the capital city.

**Methods:**

This study integrates high-resolution datasets—including the Laos Population and Housing Census, the Global Human Settlement Layer, OpenStreetMap, and Meta’s Data for Good platform—to examine dengue incidence in Vientiane from 2012 to 2018. A negative binomial regression model was employed to assess the influence of urban built-up expansion, human mobility, migration patterns, and infrastructure quality on dengue risk. Additionally, the study investigated whether structural urban risk factors remained stable across different periods dominated by distinct dengue serotypes.

**Results:**

Vientiane underwent significant urban expansion from 1990 to 2015, particularly in its periphery. Our findings reveal that recently urbanised areas with high daytime population influx exhibited the highest dengue incidence, reinforcing the role of urban centrality in shaping transmission dynamics. Migration patterns significantly influenced dengue risk, with villages hosting a larger proportion of foreign residents and Lao individuals born outside Vientiane experiencing higher incidence rates. Additionally, the availability of piped water emerged as a protective factor, as households without in-house water access were consistently associated with higher dengue incidence. Importantly, while the built-up environment and centrality played a stable role in transmission, their relative influence fluctuated with serotype changes, particularly with the emergence of *Dengue 4* in Vientiane.

**Conclusions:**

This study underscores the importance of integrating urban planning, mobility analysis, and public health surveillance to better manage infectious disease risks in rapidly expanding cities. The findings highlight the need for proactive infrastructure investments—particularly ensuring water access—to mitigate dengue risk in newly urbanised areas. Given the persistence of urban factors across different serotype-dominant periods, our results suggest that structural characteristics of the city exert a more consistent influence on dengue transmission than biological factors alone. Future research should adopt a spatiotemporal approach to refine risk models and develop more effective urban health interventions.

## Author summary

Dengue fever, a mosquito-borne disease, remains a major health concern in Southeast Asian cities. This study examines how urbanisation, mobility, and migration patterns influence dengue risk in Vientiane, Laos, between 2012 and 2018. Using high-resolution datasets, we assess how land-use change and population movements shape transmission dynamics.

Our findings reveal that dengue risk is highest in recently urbanised areas with high daytime population influx. These areas, often lacking adequate infrastructure, provide favourable conditions for *Aedes* mosquito breeding while also concentrating human activity at day, facilitating virus transmission. Migration patterns also play a role: villages with more foreign residents and Lao individuals born outside Vientiane—who may have lower immunity—reported higher dengue incidence. In contrast, villages with higher access to piped water exhibited lower dengue incidence, highlighting the role of infrastructure in disease prevention.

Importantly, while urban expansion consistently shaped dengue transmission, its influence varied across different epidemic phases, particularly with the emergence of *Dengue 4*. This suggests that urban factors interact with serotype dynamics, reinforcing the need for adaptive public health strategies. As Vientiane undergoes rapid transformation, integrating health risk assessment into urban planning will be crucial to prevent the unintended consequences of development on vector-borne disease transmission.

## 1. Introduction

Dengue fever, a vector-borne disease transmitted by *Aedes* mosquitoes, remains a major public health challenge across Southeast Asia, where it is among the leading causes of hospitalisation, particularly in children [1,2]. Urbanisation is widely recognised as a key driver of dengue transmission [3]. Yet its effects are multi-faceted and context-dependent, creating both risks and protective factors for disease spread. The expansion of urban areas can increase vector densities, alter human movement patterns, and reshape socio-environmental conditions, leading to highly heterogeneous transmission dynamics within cities.

The impact of urbanisation on vector ecology is particularly marked in built-up expansion, which modifies mosquito breeding habitats and contributes to the proliferation of synanthropic mosquitoes, such as *Aedes aegypti* and *Aedes albopictus* [4–6]. Newly urbanised areas often develop with incomplete infrastructure, poor drainage, and irregular water access, creating ideal conditions for vector breeding and intensifying dengue transmission risks. This pattern has been observed in several rapidly expanding cities, where peri-urban environments have emerged as high-risk areas for arboviruses such as dengue [7].

Beyond environmental transformations, urban socio-economic structures also shape disease exposure. While several studies have linked socio-economic deprivation to increased dengue incidence, findings remain inconsistent, likely due to the complex interplay between built environments, human mobility, and vector ecology [4,8–13]. In high-income neighbourhoods, for example, improved infrastructure may reduce mosquito breeding sites while simultaneously facilitating human movement, increasing the potential for virus dispersal [14].

Human mobility is a critical, yet often overlooked, factor in dengue epidemiology. Research has shown that individuals travelling between home, workplaces, and marketplaces contribute to localised dengue transmission at the neighbourhood scale [15,16]. However, few studies have directly integrated high-resolution mobility data into dengue risk assessments at the city level. Beyond short-term daily mobility, long-term migration also influences transmission: migrants settling in urban areas may be more vulnerable to infection if they originate from rural or lower-transmission regions, where prior exposure—and thus immunity to dengue—may be lower [16].

Despite these well-established risk factors, most studies tend to examine them in isolation, without fully considering their combined effects within a unified analytical framework. The study presented here seeks to address these complexities by investigating the spatial epidemiology of dengue in Vientiane, a rapidly urbanising capital city where vector ecology, human mobility, and urban development intersect.

Dengue has been documented in Laos since the 1950s, with major epidemics involving all four serotypes in recent years [17–19]. However, research in Vientiane—the capital and primary urban centre—remains limited, and the spatial distribution of dengue within the city is poorly understood. With an annual population growth rate of 2.6% since 2005, Vientiane presents a highly dynamic environment for the proliferation of *Aedes aegypti*. Additionally, as a central hub for both rural-to-urban and international migration [20], the city is uniquely positioned to experience heightened dengue transmission risks, making it a crucial case study for understanding the combined effects of urbanisation, mobility, and vector-borne disease dynamics.

By integrating built environment characteristics, socio-demographic factors, and the spatial structure of dengue incidence from 2012 to 2018, this research provides a comprehensive view of how urban dynamics shape disease patterns. Additionally, it explores the role of serotype circulation in shaping transmission dynamics. During 2012–2013, serotype 3 accounted for 82% of confirmed cases. In 2014, serotype 4 became predominant (55% of cases), though the overall number of infections was low. In 2015, serotype 1 dominated (85% of cases), while from 2016 to 2018, serotype 4 re-emerged (65% of cases) [19]. These shifts in serotype prevalence likely influenced immunity landscapes and transmission risks, providing an opportunity to examine the structural urban factors involved in dengue exposure, despite differences in the biological context.

## 2. Materials and methods

### Ethics statement

Ethics approval was obtained from the Lao National Ethics Committee for Health Research (N◦2018.116). Both oral and written consents were obtained from all participants, or from parent or legal guardian, when necessary.

### Dengue data

In 2012, the Institut Pasteur du Laos set up a surveillance network aiming to confirm suspected dengue cases in 15 public and private hospitals in Vientiane. This dataset covers the period between 2012 and 2018 and provides a broad view of the dengue situation across villages. Samples sent by hospitals to the Institut Pasteur du Laos were confirmed by RT-PCR and/or NS1 antigen tests. The residential addresses of the individuals’ tested positives were collected and geocoded at the village level.

### Village as the unit of analysis

The village, or *Baan* in Lao, is the smallest administrative division and serves as our primary unit of analysis for studying Vientiane’s urban and dengue dynamics. All the data of our analysis have thus been aggregated at this level. With an average of 2198 inhabitants per village, this level of aggregation allows us to capture detailed population dynamics and interactions at high resolution.

The shapefile representing village polygons was obtained from Lao Decide (https://www.k4d.la). In our analysis, we focus on villages located up to 20km from the Vientiane city centre, which correlates with the sentinel networks initiated by the Institut Pasteur du Laos. This shapefile is publicly available under the Creative Commons 4.0 CC BY license.

### The socioeconomic environment

The fourth and most recent Population and Housing Census (PHC) conducted in Laos from March 1 to 7, 2015, gathers crucial population data (number of inhabitants, age categories, migration background, etc.), housing/socioeconomic data (access to water, type of housing, etc.), and data on household assets (car, mobile phone, etc.) at the household level. Vientiane has a population of 812,037 inhabitants, residing in 481 villages. This detailed dataset provides high-resolution information and is instrumental in determining how the socioeconomic environment contributes to the spread of dengue at the city scale.

### Built environment and built-up typology

Built-up coverage was obtained from the Global Human Settlement Layer (GHSL, European Union) dataset at the 38-m resolution. To analyse the evolution of the built environment we aggregated the built-up data at the village level for 1990, 2000 and 2015. This enabled us to compare the percentages of built-up in each village of Vientiane between three different periods. We then used the percentages of built-up in 1990 to define 3 categories of villages.

The data are available at this link (Licence, Creative Commons 4.0 CC BY).

### Surface temperature

At-satellite brightness temperature was retrieved from LANDSAT 8 TIRS (band 10), applying the method described by Walawender et al. 2014 [21]. The thermal image was taken on November 18 2017 at around 6:00 PM. Land surface temperature was computed using the correction equations given by the same reference. Surface temperatures were obtained at a 38m resolution and then aggregated to the village level computing the mean temperature observed per village.

The data are available from the U.S. Geological Survey at this link.

### Urban centrality

To better understand the migrant population and its location within Vientiane, we used data from the 2015 census. This census provides us the proportion of population per village i) of foreign population ii) who are not born in Vientiane.

Urbanisation often increases mobility as urban structure is spatially heterogeneous. Area with a high urban centrality index are those areas characterized by a higher concentration of workplaces, markets, and public infrastructure than the others. High centrality locations are also hubs of human population daily commuting and are thus expected to play a key role in the epidemiological dynamics of infectious diseases at the level of a city. Here we quantified urban centrality with two proxy variables: the density of infrastructure and the ratio of daytime to nighttime population at the village level.

Density of infrastructure: to locate the main educational, religious, leisure and commercial infrastructures in Vientiane, we downloaded point-of-interest (POI) data from OpenStreetMap (OSM). These data are becoming increasingly common in geographical and sociological studies of centrality and urban change [22]. As of 1 August 2019, the OSM database contained 3,231 POIs in Vientiane. To compare centrality across villages, we tallied the POIs across different categories—education (university, schools, colleges), religion (temples, churches, etc.), recreation (bars, restaurants, etc.) and commerce (banks, markets, etc.) for each village.

This data are available at this link Licence Open Database.

The Meta’s “Data for good” datasets provide insight into the spatiotemporal variation of the users in each locality: where they sleep at night and where they work, study and shop during the day. The social-media application registered 164,770 users in Vientiane (nearly 27.5% of the total inhabitants aged 18+), which is much higher than in Southeast Asia (mean around 12%). This high penetration rate and the low gender gap (‘We are social’ report, 2020) provide reliable data to understand the spatial variation of the population in Vientiane and its high-resolution location at different time of the day. We analysed the “Facebook users’ population” dataset, which estimates the number of individuals in each of the 600x600m pixels of Vientiane every 8 hours (10:00 AM, 6:00 PM, and 2:00 AM, UTC + 7). For any given pixel, the ratio of daytime to nighttime population was calculated as 100 x *Population at 2:00 AM/ Population at 10:00 AM*. An index of centrality was then computed for each village by averaging all these ratios calculated for all the pixels within the village’s polygon boundaries between 8 and 30 June 2019.The villages were then split into 3 centrality categories based on their mean value of these ratio as so:

**category 1**: villages with at least 15% extra users during daytime compared to nighttime.
**category 2**: villages with an equal number of day/nighttime users (-15% > x < 15%).
**category 3:** villages with at least 15% fewer users during daytime compared to nighttime.

### Statistical analyses

We analysed the total number of reported dengue cases in each village over the seven-year study period, as provided by the Institut Pasteur du Laos. The number of dengue cases in each village was modeled using the following regression model:


$$Y_{i}=\beta o+\beta_{t}.T_{i}+\beta_{c}.C_{i}+\beta_{tc}.T_{i}*C_{i}+\beta_{i}.x_{i1}+...+\beta_{j}.x_{ij}+\log(p_{i})$$

where ***Yi*** represents the total number of dengue cases in village *i* over the study period, *Ti* is the typology category of village *i*, *C*_*i*_ is the centrality category of village *I*, *T*_*i*_ x *C*_*i*_ is the interaction between the two, log(*p*_*i*_) is a population size offset and *xi*_1_ to *xi*_2_ are other variables included in the multi-variable model and selected as those significant at p < 0.05 from univariable models with offset.

We started with a Poisson model from which we tested a dispersion test on the residuals using the DHARMa R package [23] that provides standardized and interpretable residuals for Poisson-type of models. If overdispersion of residuals of the Poisson model were significant at p < 0.05, we replaced the Poisson model with a negative binomial model in our analysis.

Spatial autocorrelation in the residuals was tested using Moran’s I. In case of significant spatial autocorrelation at p < 0.05, we added to the model the incidences of the neighbouring villages identified from the Queen contiguity spatial weights matrix. We first started with first order continuity and tested again the presence of spatial autocorrelation in the residuals using Moran’s I. The process was reiterated by increasing the order of the continuity index until no spatial autocorrelation was detectable in the residuals from Moran’s I.

In order to correct for potential confounding effects due to potential collinearities in the multi-variable model, the statistical significance of each variable was computed by a likelihood ratio test (LRT) between the full model and the full model without the variable in question. We used asymptotic likelihood ratio tests as implemented in the lrtest() function from the lmtest R package [24]. Because our objective was not to build a predictive model, and to facilitates easy interpretation of the coefficients, we did not transform proportion variables. For categorical variables, we selected the category with the highest mean incidence of reported dengue cases as the reference category for each categorical variable. We initially fitted a single “all-years” model (2012–2018) to identify which variables were consistently associated with dengue incidence across the entire study period. To examine whether the relationships between dengue incidence and environmental or demographic factors varied under different serotype-dominant conditions, we then split years into four successive periods based on the predominant circulating serotype as reported in [19]: 2012–2013 (DENV-3), 2014 (DENV-4), 2015 (DENV-1), and 2016–2018 (DENV-4). This classification allowed us to evaluate the robustness of predictors through time.

The data can be found on this repository: https://github.com/ecomore2/ecomore2.github.io.

## Results

### Physical, environmental and social evolution of Vientiane

**Fig 1:**
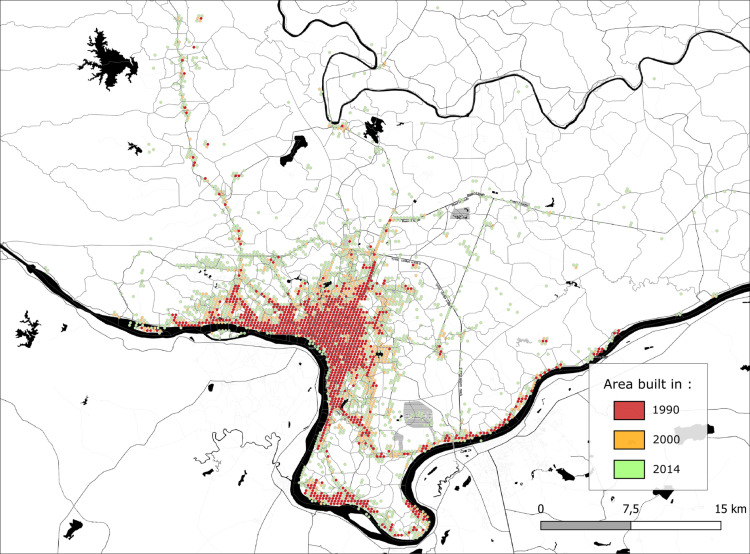
Built-up areas of Vientiane constructed in 1990, 2000 and 2014 (built = minimum 10% built-up coverage of a 100m2 grid). Map data: Global Human Settlement.

According to the 2015 national census, Vientiane’s population reached 812,037, a 16% increase from 625,000 in 2005. From 2000 to 2014, the province’s built-up surface area expanded by 20%, as detected by the Global Human Settlement Layer (GHSL), with the most notable growth occurring in Vientiane’s periphery (refer to Fig 1). Focusing on the urban part of Vientiane, our analysis of dengue’s environmental impact concentrates 544,904 inhabitants (68% of the overall population of the Vientiane region).

**Fig 2:**
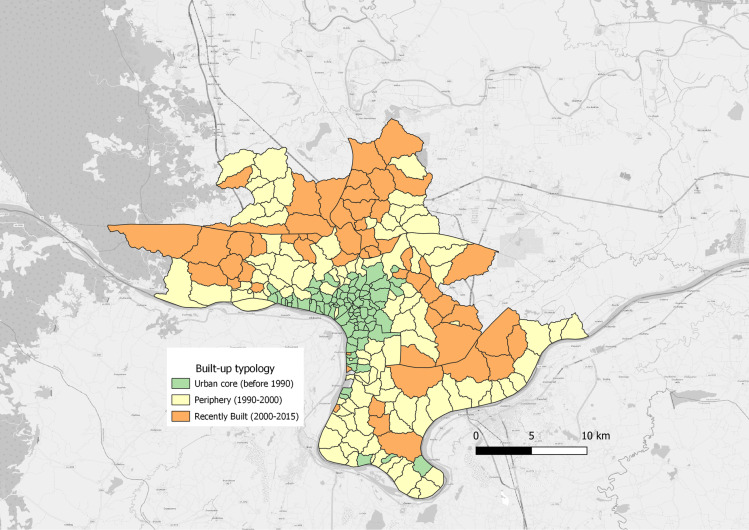
Typology of villages as identified from the GHSL dataset. Map data: Global Human Settlement, Village Layer: Lao Decide. Basemap: OpenStreetMap.

The “urban core” category includes villages with a substantial proportion of built-up area detected by GHSL in 1990 (more than 60% of the surface area). This category encompasses the centre of Vientiane and includes villages adjacent to the economic corridor in the southeast part of the city. The “periphery” comprises villages with low built-up in 1990 (10% < x < 60%) and a modest increase between 2000 and 2015 (less than 30%). The “recently urbanised” category refers to villages with low built-up in both 1990 and 2000 (x < 10%) and a significant increase in built-up areas between 2000 and 2015 (more than 30%). Typology is presented on Fig 2.

**Fig 3:**
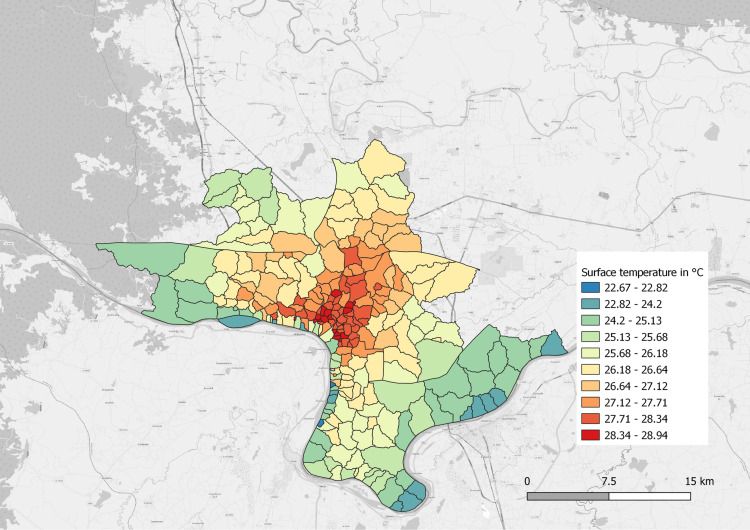
Land surface temperature detected trough Landsat 8. Map Data: Landsat 8 (Data available from the U.S. Geological Survey). Village Layer: Lao.

We observed significant variation in surface temperatures within Vientiane, ranging from 22.27 to 28.94 °C (see Fig 3). Built-up density explains a significant proportion of this variation (R² = 0.71), highlighting the strong impact of urban structures on thermal variations.

The population in the study area is characterized by heterogeneous density, ranging from 36 to 38,010 inhabitants/km². As anticipated with the built-up ratio coverage of a village, population density is higher in the urban core of the city (5,049 inhabitants/km²) and lowest in the Recently urbanised category (1,350 inhabitants/km²) (Table 1). The Urban core category is characterized by high average living area per, higher number of older residents (share of 65 + individuals) and lower economically active population than in other categories. The share of non-farm sector activity is lower in the urban core and recently urbanised categories.

**Table 1:** Built-up typology of Vientiane and mean percentage of household asset, of international and national migrants in Vientiane and points of interest (POIs) and ratio of Meta users registered at day.

			Urban core	Periphery	Recently urbanised
**Built-up evolution and surface temperature**	N		86	107	49
	Share of the village covered with built-up	In 1990	74.90%	26.00%	6.30%
		in 2000	79.00%	34.90%	13.30%
		in 2015	82.60%	41.50%	31.80%
	Mean LST in°C	18/11/2017	27.41	26.02	24.21
**Population and household asset**	Total population		152,504	240,743	138,712
	Mean population per village		1914.4	2264.9	2708.2
	Share of total population		29.40%	43.30%	27.90%
	Average living area per person (in m^2^)		38	24.1	23.4
	Population density (persons/km^2^)		5049.8	1700.4	1350.4
	Percentage of population aged 65 years and older		5.6%	4.4%	3.5%
	Percentage of literate population		97.8%	97.1%	97.5%
	Percentage of economically active population		31.1%	28.7%	35.5%
	Percentage of population with main activity unemployed		2.4%	1.9%	2.4%
	Percentage of households with farmland		28.1%	35.9%	26.1%
	Percentage of population with main activity non-farm sector		96.9%	79.6%	90%
	Percentage of households having electricity		98%	98.4%	98.6%
	Percentage of households with water access in-house		92.6%	94%	91.6%
	Percentage of houses with concrete roof		20.3%	21.3%	20.4%
**Urban centrality**	Mean ratio of Meta users at day (100 = night)		123.1	97.9	102.3
	Number of point of interest per 1,000 individuals		5.9	1.8	0.4
	Numbers of point of interest	Total	657	195	47
		Tourisms	245	78	22
		Schools	89	28	11
		Temples	62	32	6
		Cafes	180	35	1
		Banks	72	18	2
		Parcs	5	2	2
		Universities	1	2	1
		Colleges	8	2	4
**Migration**	Percentage of individual born outside Vientiane		29.8%	23.5%	33.3%
	Percentage of foreigners		6.1%	1.4%	1.1%

Source: Lao Statistic Bureau, 2015 ** OpenStreetMap and Meta for good, 2020.

**Fig 4:**
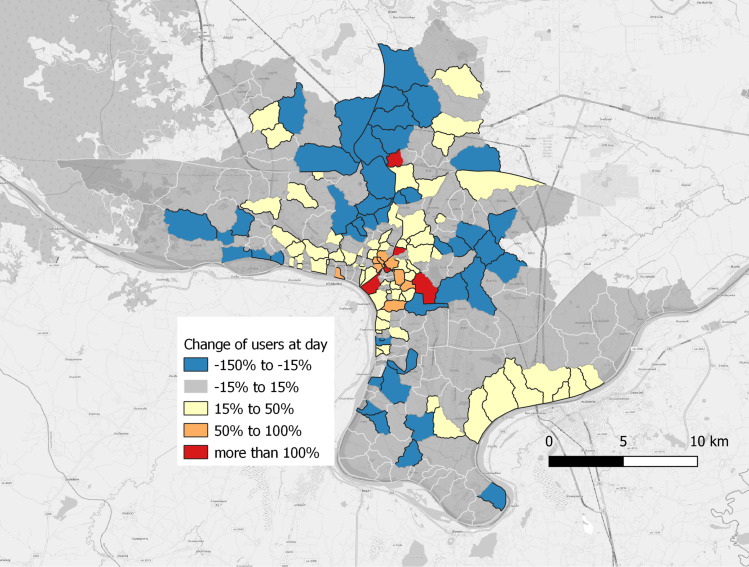
Difference in number of Meta users registered during daytime vs nighttime hours. Map Data: Data for Good @Meta data.

The OpenStreetMap dataset reveals a greater number of café, banks and tourism infrastructures in the urban core. Schools, temples and colleges appear to be more unevenly distributed between categories (Table 1). Centrality wise, the villages were distributed across the three centrality categories as follows: 79 villages in the More users at day category, 128 villages in the Equal category, and 42 villages in the Less users at day category. The urban core category has a much higher ratio of individuals at day, while the two others have comparable numbers of users at day and night. Fig 4 shows the ratio of users at day versus night in all the studied area.

The crude birth rate in the Vientiane Capital district, as reported in the 2015 census, is the lowest in Laos with 22 births per 1000 inhabitants. The percentage of non-Lao individuals is higher in the urban core (6.1% of the population) compared to the urban periphery (1.4%) and the recently urbanised category (1.1%) (see Table 1). Approximately 10,000 inhabitants in Vientiane hold non-Lao citizenship. The percentage of the Lao population not born in Vientiane is higher in the urban core. and the recently urbanised periphery (32.4% and 36.3%, respectively) in comparison with the first periphery (23.5%).

### Spatio-Temporal evolution and the geography of dengue incidence in the city of Vientiane

A total of 2,837 dengue cases were recorded in the urban area of Vientiane between 2012 and 2018, of which we could locate only 2,429 (i.e. 85% of all cases). Dengue outbreaks in Vientiane typically peak during the rainy season (May to January), following a period of lower or moderate DENV transmission in the dry season. The largest epidemic occurred in 2013, when 948 cases were confirmed in the capital city, accounting for 39% of cases recorded between 2012 and 2018.

**Fig 5:**
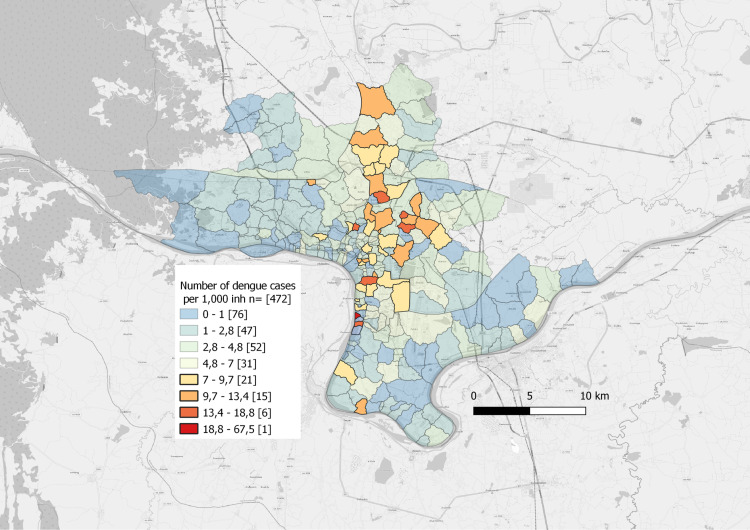
Dengue incidence rate detected per village (2012-2018) Map Data: Institut Pasteur du Laos. Village Layer: Lao Decide.

The seven years incidence within villages is going from zero to 61 cases per 1,000 inhabitants (mean = 4.35). Dengue incidence in Vientiane shows significant spatial variation, with the highest cumulative rates observed in the northern and central districts of the city. This distribution may be influenced by urban density and socioeconomic factors. The West and the South-East parts tend to gather villages with low incidence of dengue (Fig 5). Cumulative incidences are higher in the recently urbanised villages followed by the villages of the urban core of Vientiane and the 1st periphery (Table 2).

**Table 2:** Incidence of dengue cases registered during the different years per SE category (per 1,000 inh).

Category	2012	2013	2014	2015	2016	2017	2018	Cumulative incidence per category
Urban core	0.32	1.74	0.04	0.38	0.40	1.27	0.27	4.42
Periphery	0.19	1.29	0.01	0.32	0.29	1.29	0.24	3.63
Recently urbanised	0.29	2.29	0.01	0.44	0.51	1.52	0.35	5.42
Mean	0.26	1.70	0.02	0.37	0.38	1.35	0.28	4.35

### Uni-variable analyses

The result of the dispersion test realised on the Poisson regression showed a p-value < 0.05 indicating a significant overdispersion, leading us to adopt a Negative Binomial model. The non-spatial version of the model showed significant spatial autocorrelation, which was resolved by incorporating incidence data from first-order neighbours.

We analysed the association between dengue cases in a village during the seven years under investigation with log(population) as an offset and using the newly built category with more individual at day as reference, since all other categories had lower cumulative incidence (Table 3A).

**Table 3:** Results of the uni-variable negative binomial regressions: A) Built-up and centrality categories and their interaction. B) Population and household asset. C) Centrality profile. D) Migration variables. log of population as offset. * = p-value below 0.05.

A. Built-up, centrality and their interaction	Coef.	B. Population and household asset	Coef.
**Urban Core**		Percentage of household under poverty line	–0.87*
**More users at day**	**–1.09**	**Average living area per person**	0.18*
**Equal**	–1.32*	**Population density (Persons/km2)**	.052*
**Less users at day**	–1.58*	Percentage of population aged 65 years and older	0.36
**Periphery**		Percentage of literate population	0.12
**More users at day**	–1.60*	**Percentage of economically active population**	0.20*
**Equal**	–1.43*	Population with main activity unemployed	0.02
**Less users at day**	–1.59*	Percentage of households with farmland	–0.23
**Recently urbanised**		**Percentage of population with main activity non-farm sector**	–0.13*
**More users at day**	**REF**	Percentage of households having electricity	0.03
**Equal**	–1.60*	**In-house water**	–0.7*
**Less users at day**	–1.31*	House with concrete roof	.36
**C. Migration**	**Coef.**	**D. Centrality**	**Coef.**
**Share of individual born outside Vientiane**	0.18*	**Point of interest**	
**Share foreigner**	0.20*	Total	0.23
		Hotel	0.11
		Schools	0.05
		Temples	0.18
		Restaurants	0.36
		Banks	–0.04
		University	0.11
		Colleges	0.35
		Park	–0.23

Land surface temperature did not have any impact on the cumulative incidence of dengue.

Significant positive relationships were identified for the average living area per person, population density, the percentage of economically active population, indicating that increases in these variables are associated with an increase in the dengue incidence. Conversely, a significant negative relationship was observed for the population with the main activity unemployed and the presence of in-house water supply.

Several variables, however, did not show a significant association in this analysis. These include the percentage of the population aged 65 years and older, the percentage of literate population, the percentage of households with farmland, the percentage of households having electricity, and the presence of a concrete roof (Table 3B).

Next, we assessed national and international migrations by examining continuous variables: 1) the number of non-Lao and 2) the number of individuals born outside Vientiane Capital. Both variables were significantly associate with dengue cases (Table 3C).

The recently built village with more users at day was associated with higher number of cases compared to other types of villages. It is to note the number of POI detected by OSM was not associated with dengue incidence (Table 3D).

### Multi-variable analysis

Many variables related to population, household assets, and migration that were significant in the univariable model became non-significant in the multivariable analysis. However, typology of built-up/centrality remained significant, along with the share of households with water access, the share of foreigners, and the share of Lao individuals born outside Vientiane (Table 4).

**Table 4:** Result of the multi-variable negative binomial regression considering only the significant variables from the univariable models and using the log of population as offset. P-Values = likelihood ratio test. * = p-value below 0.05.

Variables	Coef.	P > z
**Typology and Centrality profile**		
**Urban Core**		
More users at day	**-1.41**	**0.06**
**Equal**	**-1.41**	**0.06**
**Less users at day**	**-1.57**	**0.246**
**Periphery**		
**More users at day**	-1.58*	**0.046**
Equal	-1.33	0.051
**Less users at day**	-1.48*	**0.042**
**Recently urbanised**		
**More users at day**	**REFERENCE**	
**Equal**	-1.67*	**0.004**
Less users at day	-1.39	0.131
**Population and household asset**		
**In-house water**	-1.21*	**0.014**
**Migration**		
**Share of foreigner**	2.67*	**0.000**
**Share of Lao individual born outside Vientiane**	1.46*	**0.000**
**Local incidence**		
**Neighbouring incidence**	0.10*	**0.003**

The non-spatial version of the model showed significant spatial autocorrelation, which was resolved by incorporating incidence data from first-order neighbours.

As in the uni-variable analyses, the dispersion test conducted on the multivariable Poisson regression returned a p-value < 0.05, indicating significant overdispersion. Consequently, we adopted a Negative Binomial model to better account for variance inflation in the data.

Our results show that recently urbanised areas with high daytime population density faced a risk of dengue transmission significantly higher than all the other categories. Beyond these risk factors, not having water in the house remained significantly associated with higher dengue incidence (estimate = -1.21, P > z = 0.014). When expressed as an Incidence Rate Ratio (IRR), a 10% increase in internal water access corresponds to an 11% reduction in dengue incidence. Similarly, villages with a higher share of foreigners exhibited significantly higher dengue incidence (estimate = 2.67, P > z = 0.00). A 10% increase in the proportion of foreigners was associated with a 30% increase in dengue incidence

Villages with a higher share of Lao individuals born outside Vientiane also reported significantly higher dengue cases (estimate = 1.46, P > z = 0.000). Here, a 10% increase in this population share corresponded to a 16% increase in dengue incidence. Similarly, the share of foreigners in one unit had a positive association with dengue incidence.

### Spatio (I)-temporal pattern of dengue incidence

The year 2014 presented a unique challenge in the analysis due to the low number of reported dengue cases, which limited statistical power and the ability to detect meaningful associations. During this period, serotype 4 was dominant, accounting for 55% of cases, but overall transmission levels were significantly lower compared to other years. Furthermore, no variables reached statistical significance in the multivariate model, and no clear spatial patterns emerged (Table 5).

**Table 5:** Coefficient of the multivariable negative binomial regression per period using the log of population as offset and P-values from the likelihood ratio test. * = P value below 0.05 ** = P value below 0.1.

Variables	2012-2013	2014	2015	2016-2018
	**Coef and (P-values)**			
**Predominant serotype**	**Dengue 3**	**Dengue 4**	**Dengue 1**	**Dengue 4**
**Typology and Centrality profile**				
**Urban Core**				
**More users at day**	-1.29 * (0.012)	15.3 (1)	-3.16 * (0.002)	-0.97 * (0.037)
**Equal**	-1.49 * (0.004)	15.4 (1)	-2.32 * (0.004)	-0.78 (0.067)
**Less users at day**	-1.42 (0.413)	-22.29 (1)	-0.89 (0.999)	-0.89 (0.695)
**Periphery**				
**More users at day**	-1.75 * (0.031)	4.7 (1)	-0.82 (0.01)	-0.82 (0.335)
**Equal**	-1.45 * (0.07)	13.5 (1)	-2.85 (0.06)	-0.75 (0.442)
**Less users at day**	-1.46 * (0.049)	-0.89 (1)	-2.91 * (0.002)	-0.96 (0.151)
**Recently urbanised**				
**More users at day**	**REFERENCE**			
**Equal**	-1.72 * (0.004)	14.97 (1)	-3.96 (0.08)	-1.10 * (0.041)
**Less users at day**	-1.21 (0.343)	-1.7 (1)	-3.37 * (0.001)	-1.09 (0.084)
**Population and household asset**				
**In-house water**	-0.48 (0.392)	-1.73 (0.254)	-2.19 * (0.018)	-1.64 * (0.003)
**Migration**				
**Share of foreigner**	1.13 (0.316)	-4.49 (0.506)	4.18 (0.096)	-3.50* (0.028)
**Share of Lao individual born outside Vientiane**	1.28 * (0.000)	2.03 (0.108)	3.06 * (0.000)	2.15* (0.000)
**Local incidence**				
**Neighbouring incidence**	0.23 * (0.000)	4.99 (0.285)	1.55 * (0.000)	0.14* (0.029)

Overall, the multivariate analysis across all periods revealed consistent associations between dengue incidence and key environmental and demographic factors. The built-up and centrality profile displayed significant negative coefficients in most years, indicating lower dengue incidence in areas outside recently urbanised zones with high daytime activity. While effect sizes varied, the overall association between urban structure and dengue risk remained stable over time, with differences from 2016 to 2018, when serotype 4 was predominant. Indeed, several types of space in the “urban core” and “periphery” remained above the level of significance.

Migration-related factors were also significantly associated with dengue incidence. The proportion of Lao individuals born outside Vientiane consistently showed a positive association with dengue incidence with the strongest effect in 2015 when serotype 1 was predominant (3.06, p = 0.000). The proportion of foreigners had a positive effect in 2015 (4.18, p = 0.096) but a negative effect in 2016–2018 (-3.50, p = 0.028).

Neighbouring incidence was positively associated with dengue cases, though its effect was more moderate compared to other factors. The share of in-house water had protective effect against dengue incidence. Significant negative associations were found from 2015 to 2018.

## Discussion

This study aimed to investigate how urban characteristics influenced dengue transmission in Vientiane between 2012 and 2018. By leveraging a high-resolution urban dataset that captured population movements, migration patterns, and built-up expansion, we were able to track how the city evolved alongside dengue transmission dynamics. Unlike most studies that rely on static representations of urban environments, our approach incorporated diachronic urban growth and population geography allowing for a more nuanced understanding of spatial and temporal risk factors.

### Water access and the role of infrastructure in dengue mitigation

Unlike socioeconomic deprivation, which was not a significant predictor, water access appeared as a key determinant of dengue risk. Areas where fewer households had direct water access displayed consistently higher incidence, particularly in 2015 (-2.19, p = 0.053) and 2016–2018 (-1.64, p = 0.006). These results suggest that reliance on stored water in open containers—common in areas with inadequate water supply—provides additional breeding sites for *Aedes* mosquitoes, as reported in Delhi [25,26] and Dhaka [27].

This indicates that urban infrastructure, particularly improved water distribution systems, plays a crucial role in reducing dengue risk, independent of household income levels. Unlike cities such as Santa Cruz de la Sierra [28], where high-income areas experience dengue outbreaks due to vector ecology and human mobility, our findings suggest that in Vientiane, infrastructure deficiencies, rather than economic disparities, contribute to vector proliferation.

### Migration and local immunity dynamics

Interestingly, the proportion of foreigners in a village showed contrasting effects, being positively associated with incidence in 2015 (4.18, p = 0.096) but negatively associated in 2016–2018 (-3.50, p = 0.052). This shift could be linked to changing exposure patterns and immunity landscapes among different migrant populations. These results reinforce the need for better integration of mobility and immunity data into dengue risk assessments.

Migration patterns had a strong and consistent association with dengue risk. The share of Lao individuals born outside Vientiane was significant in multiple models, peaking in 2015 (3.06, p = 0.000) and remaining significant in 2016–2018 (2.15, p = 0.000). While this pattern could initially suggest differences in local immunity—where migrants from lower-transmission areas may be more susceptible—recent studies indicate that dengue incidence rates are now comparable between rural and urban settings [29]. Rather than a simple urban-rural gradient, the elevated dengue incidence among migrant populations may instead reflect differences in exposure to multiple serotypes in hyperendemic urban environments like Vientiane. The rapid co-circulation of serotypes in the capital, as demonstrated by surveillance data, could result in increased secondary infections and more severe disease outcomes for newcomers who may not have been previously exposed to the full spectrum of circulating serotypes.

### Urban expansion and human mobility drive dengue transmission

A growing body of literature highlights the role of human mobility in sustaining dengue transmission. In Sri Lanka, research has demonstrated that travel patterns exceeding mosquito flight range drive virus dispersal [30]. In Vietnam, dengue transmission dynamics have been linked to the interplay between climate change, infrastructure, and population mobility [31]. Study in Pakistan [32] has shown that integrating mobility data improves dengue forecasting models, reinforcing that human-driven virus movement complements vector-driven transmission cycles. At the city level, studies from Bangkok [33], Fortaleza [34] and Singapore [35] have demonstrated that public transport and high human connectivity facilitate the spread of dengue.

The transformation of peripheral urban spaces into fully urbanised environments has already demonstrated its capacity to create ecological conditions conducive to mosquito abundance, particularly for *Aedes aegypti* [36,37]. Newly urbanised areas, often characterised by incomplete infrastructure, standing water accumulation, and increased human mobility, provide favourable breeding conditions for *Aedes* mosquitoes, reinforcing the need for integrated vector control strategies.

Our findings indicate that recently built areas with high centrality exhibited a higher risk throughout the entire study period (2012–2018), highlighting the importance of their interaction in shaping dengue dynamics. Newly developed urban spaces likely altered mosquito abundance and species diversity, facilitating breeding sites, while centrality may have amplified viral transmission by increasing human exposure to infected mosquitoes. However, further research is needed to confirm these patterns, both in Vientiane and in other contexts where peri-urbanisation is becoming a dominant model of urban development. Indeed, the construction of these areas is part of broader urban development strategies promoted by local and national authorities. They aim to create new peripheral zones that accommodate Vientiane’s rapid expansion while decentralising economic, administrative, and commercial activities traditionally concentrated in the city centre. However, the epidemiological impact of these emerging urban areas appears to have been underestimated, highlighting the need for an integrated approach to urban planning and public health interventions.

### Temporal variations and serotype influence

Despite the consistency of these factors in the overall model, their significance evolved across different epidemic phases, suggesting shifts in transmission dynamics. During phases dominated by Dengue 4 (2014, 2016–2018), the significance of urban typology and centrality weakened. Instead of large-scale urban spatial factors, intra-village conditions such as household water access and migration patterns became more prominent predictors of dengue risk.

Recent studies suggest that Dengue 4 was newly introduced into the population, which may explain this shift. The emergence of a novel serotype could have altered transmission patterns, increasing the importance of localised environmental and individual-level factors over broader urban mobility structures. However, the continued significance of ‘the share of individual born outside Vientiane’ suggests that certain population groups—linked to past exposure patterns or differential immunity—remained consistently vulnerable.

While urban typology was a strong predictor in earlier years, our findings indicate that its significance declined over time, particularly with the emergence of Dengue 4. This suggests that urban expansion alone is insufficient to explain dengue risk unless combined with other environmental conditions such as inadequate water infrastructure. This aligns with previous research demonstrating that urbanisation impacts dengue risk most when coupled with poor sanitation and water management.

### Dengue and the role of climate

Dengue risk can be influenced by meteorological conditions such as rainfall and temperature, which play crucial roles in determining mosquito abundance and their capacity to transmit the virus [38]. Due to the inadequate spatial resolution of the available data and the high resolution of village-level analysis, we could not integrate information such as rainfall or air temperature into this study. In Delhi, other studies have highlighted a relation between reproduction rate of dengue and human density and land surface temperature (LST) [39]. However, in Vientiane, we found no significant association between LST and dengue incidence. We suggest that the weak correlation between urbanisation quality, LST, and virus incidence can be attributed to the different climatic context. In Delhi, mosquito geography is highly influenced throughout the year by the presence of low temperatures that inhibit *Aedes aegypti* reproduction during winter, except within urban heat islands. Conversely, Vientiane’s consistently warmer temperatures throughout the year facilitate continuous mosquito breeding across the entire city. Surface temperatures play a far less significant role in mosquito reproduction in a tropical climate like Vientiane’s as compared to a continental climate like Delhi’s, where temperature fluctuations are more pronounced.

## Conclusion

Our findings demonstrate the consistency of key urban risk factors for dengue transmission in Vientiane, despite variations in dominant serotypes and epidemic conditions. Across all periods, dengue incidence was consistently associated with urban typology, mobility patterns, and infrastructure availability, highlighting that the structural characteristics of the city exert a stronger influence on disease risk than serotype fluctuations. Newly urbanised areas with high daytime population influx emerged as persistent hotspots, reinforcing the role of centrality and human mobility in sustaining dengue transmission. Furthermore, households lacking direct water access consistently faced higher dengue incidence, likely due to increased reliance on stored water, which serves as a breeding site for *Aedes* mosquitoes.

Beyond individual risk factors, our findings underscore the interplay between urban development, migration, and vector ecology in shaping disease transmission. Rapid urban expansion and economic restructuring have led to the creation of peripheral urban zones, originally intended to ease pressure on the city centre, but which have inadvertently become high-risk areas for dengue transmission. The transformation of peripheral areas into fully urbanised environments alters mosquito habitats, increases human exposure to vectors, and reshapes population immunity dynamics, contributing to long-term changes in dengue epidemiology.

Despite these key findings, we observed temporal variations in risk factors, particularly with the introduction of Dengue 4 from 2015, which altered the significance of urban typology and centrality combined. This suggests that the impact of urbanisation on dengue transmission is contingent on both environmental conditions and circulating serotypes.

While climate factors such as temperature and rainfall are known to influence mosquito abundance, we found no significant association between land surface temperature (LST) and dengue incidence in Vientiane. Unlike in cities with strong seasonal temperature variations (e.g., Delhi), Vientiane’s consistently warm climate likely supports mosquito breeding throughout the year, making other environmental and socio-spatial factors more relevant in shaping dengue risk.

Future research should further explore the interactions between land-use change, mobility, and vector ecology, while incorporating longitudinal and climate data to enhance risk prediction in evolving urban environments. Expanding the temporal dimension of analysis—for instance, through monthly-level studies or finer-scale spatiotemporal modelling—could help identify seasonal variations and outbreak triggers more precisely. Similarly, integrating high-resolution mobility datasets and climatic fluctuations would provide a more dynamic understanding of how human movement and environmental conditions shape dengue transmission patterns over time.

This study represents a first step in understanding the relationship between urbanisation, mobility, and dengue transmission in Vientiane. While our findings highlight key structural and demographic risk factors, a more detailed spatiotemporal approach would improve predictive modelling, enabling more targeted public health interventions and better urban planning strategies to mitigate dengue risk in rapidly expanding cities. By incorporating these refinements, future studies could bridge the gap between epidemiological modelling and urban policy, ensuring that dengue risk management is not only reactive but also anticipatory, adapting to the evolving socio-environmental landscape of rapidly growing cities.

Indeed, these findings highlight the urgent need for proactive urban planning to accompany Vientiane’s rapid expansion. Ensuring that newly developed urban zones are equipped with adequate infrastructure—particularly piped water access—will be essential for mitigating long-term risks. Beyond Vientiane, these dynamics reflect broader regional trends in Southeast Asia, where peri-urban expansion is reshaping dengue transmission patterns. Cities such as Bangkok, Ho Chi Minh City, and Yangon face similar mobility-driven disease risks, reinforcing the need to shift from reactive dengue control strategies to forward-thinking urban health planning. Rather than relying solely on short-term vector control, cities must integrate disease risk management into broader infrastructure and mobility policies, ensuring that urbanisation does not inadvertently amplify infectious disease spread.

## Limitation

One significant limitation is to not capture the mobility of individuals under 18 years old, a limitation not unique to Meta but also applicable to data from mobile phone users. Another significant limitation is the absence of vector data with sufficient spatial and temporal coverages, which is crucial for understanding the relationship between mosquito populations and dengue incidence. The lack of detailed entomological data prevents the estimation of a direct relationship between mosquito abundance or species distribution and disease incidence. To address this gap, it is essential for public health authorities to establish regular, systematic vector surveillance. Comprehensive vector data collection will enable more accurate analysis of dengue transmission dynamics, providing critical insights for targeted vector control interventions.

Another limitation is the incomplete integration of meteorological spatial variations, particularly due to the granularity of available dengue incidence data. While rainfall and temperature are well-documented drivers of vector dynamics and dengue outbreaks, the spatial scale of available meteorological data did not always align with the village-level resolution of dengue cases, limiting our capacity to establish precise climate-disease relationships. High-resolution climate datasets such as soil humidity would enhance the understanding of how local microclimates influence vector populations and virus transmission.

Furthermore, the lack of granular, high-frequency epidemiological data beyond census-derived indicators presents challenges for integrating multiple datasets. To improve future studies, strengthening longitudinal surveillance with finer spatial resolution would allow for a more comprehensive integration of mobility, environmental, and socio-demographic factors in dengue transmission models.
